# Development and Validation of an HPLC-MS/MS Method for Rapid Simultaneous Determination of Cefprozil Diastereomers in Human Plasma

**DOI:** 10.1155/2018/6959761

**Published:** 2018-09-13

**Authors:** Guodong He, Liping Mai, Xipei Wang

**Affiliations:** Medical Research Center, Guangdong General Hospital, Guangdong Cardiovascular Institute, Guangdong Academy of Medical Sciences, Guangzhou 510080, China

## Abstract

**Background:**

Both* cis*- and* trans*-cefprozil have antimicrobial activity, but their potencies are quite different. It is therefore necessary to develop a sensitive method to simultaneously determine both isomers for pharmacokinetic and bioequivalence studies.

**Methods:**

An LC-MS/MS method, using stable isotope-labeled cefprozil as the internal standard, was developed and validated. The analytes were extracted from plasma by protein precipitation and separated on a reverse-phase C_18_ column using a gradient program consisting of 0.5% formic acid and acetonitrile within 4 min. The mass spectrometry acquisition was performed with multiple reaction monitoring in positive ion mode using the respective [M+H]^+^ ions,* m/z* 391.2→114.0 for cefprozil and 395.0→114.5 for cefprozil-D4.

**Results:**

The calibration curves were linear over the ranges of 0.025–15 *μ*g/mL for* cis*-cefprozil and 0.014–1.67 *μ*g/mL for* trans*-cefprozil. The accuracies for the* cis* and* trans *isomers of cefprozil were 93.1% and 103.0%, respectively. The intra- and interassay precisions for the QC samples of the isomers were < 14.3%. The intra- and interassay precisions at the LLOQ were < 16.5%.

**Conclusions:**

The method was sensitive and reproducible and was applied in a pilot pharmacokinetic study of healthy volunteers.

## 1. Introduction

**Figure 1:**
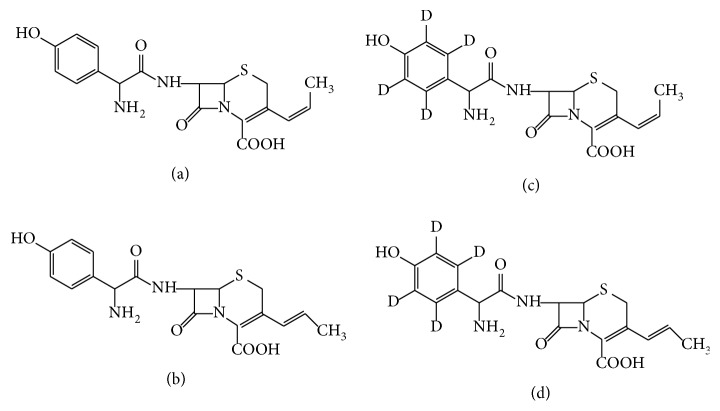
Chemical structures of* cis*-cefprozil (a),* trans*-cefprozil (b),* cis*-cefprozil-D4 (c), and* trans*-cefprozil-D4 (d).

Cefprozil is a second-generation oral *β*-lactam in the cephem class. It is composed of* cis* (BMY-28100) and* trans* (BMY-28167) isomers in an approximately 9:1 ratio [1–3] (Figure 1). It has a broad* in vitro* spectrum of antimicrobial activity against both Gram positive and Gram negative bacteria and is penicillinase resistant [4–12]. Both isomers exhibit antimicrobial activity [1–4]. The pharmacological activity of resistant Gram positive bacteria, streptococcus, and staphylococcus activity of the* cis* isomer is equal to the* trans* isomer; however, the pharmacological activity of resistant Gram negative bacteria of the* cis* isomer is eight times that of the* trans* isomer [8]. It is therefore necessary to develop a sensitive method to determine both isomers for pharmacokinetic studies and effective management of therapy. High-performance liquid chromatography-ultraviolet (UV) spectrophotometric methods for the determination of cefprozil diastereomers in human plasma, separately or simultaneously, have previously been reported [12–17]. There are, however, some limitations of these methods, including long analysis time and complex mobile phase compositions. In addition, there are limitations that arise from the use of UV detection, such as endogenous peak interference and a lower limit of quantification (LLOQ) that does not meet the requirements for clinical pharmacokinetic studies. As the technology has developed, LC-MS/MS has been universally applied for analysis of multiple analytes [18–21]. An LC-MS/MS method for simultaneous determination of cefprozil diastereomers in human plasma has recently been reported. The method used an electrospray ion source in negative ion mode with cephalexin as internal standard (IS) and achieved LLOQ values of 0.125 and 0.04 *μ*g/mL for* cis*- and* trans*-cefprozil, respectively [22]. However, it is not appropriate to use cephalosporins as internal standards because they are unstable. For mass spectrometry-based assays, a stable isotope-labeled IS is generally preferred. Moreover, we found that better sensitivity was achieved in the determination of cefprozil using an electrospray ion source in positive rather than negative ion mode. In the published methods, at least 250 *μ*L of plasma sample was required for protein precipitation using acetonitrile. Because the level of* trans* isomer is only about 10% of total cefprozil, we developed a high-performance reverse-phase liquid chromatography-positive ion electrospray tandem mass spectrometry method using isotope-labeled IS that gave a lower LLOQ compared to the previously published LLOQ values using negative ion mode. This method required only 100 *μ*L plasma sample prepared by protein precipitation using methanol. In this study, we focused on the development of a new validated method for simultaneous determination of cefprozil isomers in plasma, enabling effective management of therapy.

## 2. Experimental

### 2.1. Reagents and Chemicals

Reference standards of cefprozil (*cis* isomer 85.9%,* trans* isomer 9.2%) were purchased from the National Institutes for Food and Drug Control. The cefprozil-D4 (Figure 1) indicates the positions at which cefprozil was labeled;* cis* isomer 89.9% and* trans* isomer 9.4% were purchased from TLC Pharmaceutical Standards Ltd. (Aurora, Canada). HPLC grade formic acid (a minimum of 99.0% purity) was obtained from Dikma Technologies Inc. (CA, USA). HPLC grade acetonitrile (ACN) and methanol (MeOH) were obtained from Merck KGaA (Darmstadt, Germany). Water was purified using a Labscale tangential flow filtration system (Milli-Q RG, Millipore, from Merck KGaA). Drug free (heparin) plasma was obtained from Guangdong Academy of Medical Sciences (Guangzhou, China).

### 2.2. Equipment

The HPLC system consisted of a SIL-20AC autosampler, an LC-20AB pump, and a CTO-20A column oven (Shimadzu, Kyoto, Japan). A reverse-phase Gemini C_18_ column, 150 × 2.0 mm, i.d. 3 *μ*m (Phenomenex, Torrance, CA, USA), was used to separate the diastereomers. A C_18_ column, 4 × 2.0 mm (Phenomenex), was used as a guard column. The HPLC was connected to a triple quadrupole 4000QTRAP mass spectrometer (Applied Biosystems, Foster City, CA, USA) equipped with a heated electrospray ionization source. An HS602 rotary vacuum pump (VARIAN, USA) and an NM32LA nitrogen generator (Peak scientific, Renfrewshire, Scotland, UK) were used. Analyst 1.4.2 software was used for optimization of tuning parameters, data acquisition, and processing.

### 2.3. Liquid Chromatography Conditions

The mobile phase consisted of 0.5% formic acid (mobile phase A) and ACN (mobile phase B). The gradient was started at 5% B, linearly increased to 20% B during 1.4 min, maintained for 1.5 min, increased to 70% B during 0.1 min, and then maintained for 0.5 min to wash out interference. The mobile phase was then returned to 5% B for reequilibration. The flow rate was 0.3 mL/min and the total run time was 4 min. The column oven was maintained at 25°C and the autosampler needle rinsing solution consisted of methanol:water (50:50, v/v). The injection volume was 3 *μ*L for each sample.

### 2.4. Mass Spectrometry Conditions

**Figure 2:**
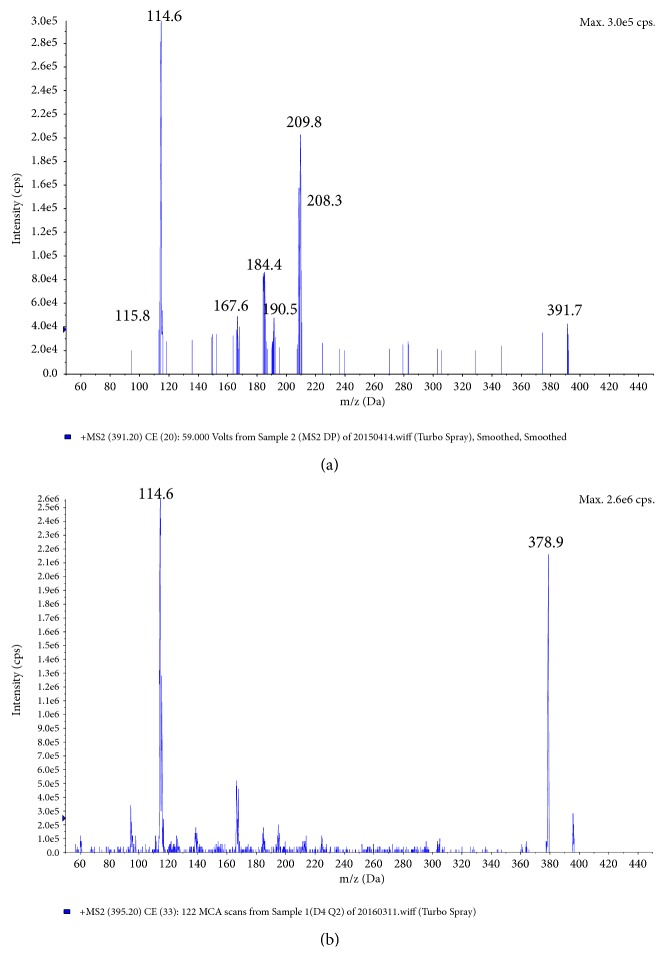
Product ion scans of cefprozil (a) and cefprozil-D4 (b).

The mass spectrometer was operated in positive ion mode with multiple reaction monitoring (MRM). The API-4000 QTRAP was set up with the following optimized conditions for the target analytes: curtain gas, 30 psi; ion source gas, 1; and gas 2 settings of 30 and 60 psi, respectively; collision activated dissociation level setting of medium; heater gas temperature of 600°C; and ion spray needle voltage of 5000 V. Nitrogen was used as the heater gas, nebulizing gas, CAD gas, and curtain gas. The monitored [M+H]^+^ ions were* m/z* 391.2→114.0 for* cis*- and* trans*-cefprozil, and* m/z* 395.0→114.5 for* cis*- and* trans*-cefprozil-D4, with collision energies of 30 eV and a declustering potential of 70 V. The dwell time was set to 200 ms per ion pair. The product ion scans for cefprozil and cefprozil-D4 are shown in Figure 2.

### 2.5. Preparation of Quality Control and Calibration Standard Samples

Standard solutions (1 mg/mL) of cefprozil and cefprozil-D4 were prepared in methanol and stored at -20°C. Intermediate dilutions of cefprozil (200 *μ*g/mL) were in methanol:water (50:50, v/v). A working solution of cefprozil-D4 (30 *μ*g/mL), routinely used as the IS solution, was diluted in methanol:water (50:50, v/v). Standard solutions of cefprozil in human plasma were prepared by spiking with an appropriate volume (< 10 *μ*L/mL) of the diluted stock solutions, giving final concentrations of 0.025, 0.05, 0.125, 0.25, 0.5, 1, 5, 10, 12, and 15 *μ*g/mL of* cis*-cefprozil and 0.014, 0.028, 0.056, 0.112, 0.556, 1.112, 1.33, and 1.67 *μ*g/mL of* trans*-cefprozil. Quality control samples were prepared at three different nominal concentrations: 0.05, 4.1, and 12.4 *μ*g/mL for the* cis* isomer; 0.028, 0.445, and 1.33 *μ*g/mL for the* trans* isomer.

### 2.6. Sample Extraction

Plasma samples (100 *μ*L) were transferred into 1.5-mL Eppendorf tubes. A solution of cefprozil-D4 (20 *μ*L, 30 *μ*g/mL) was added to the tubes and vortexed for 10 s. Methanol:0.1% formic acid (100:0.1, v/v, 400 *μ*L) was added to each of the tubes. The samples were vortex-mixed for 2 min and centrifuged at 12,000 × *g* for 10 min at 4°C. Samples of the supernatants (3.0 *μ*L) were used for analysis.

### 2.7. Validation Procedure

The method was validated according to the guidance for bioanalytical method validation [23].

Every batch, containing calibration standards, was analyzed on two different days. In each batch, six replicates of the LLOQ, low, medium, and high QC samples were prepared to evaluate intra- and interday precision and accuracy. The acceptance criteria for accuracy should be within ± 15% (LLOQ within ± 20%) and for precision should be ≤ 15% (LLOQ ≤ 20%) relative standard deviation [23].

Six randomly selected human blank plasma samples were used to test specificity. The samples were processed according to the sample preparation procedure and injected into the HPLC-MS/MS system to determine the extent to which endogenous components in plasma influenced the retention time of cefprozil and the IS.

Linearity was determined from calibration curves of peak area ratio of standard cefprozil to cefprozil-D4 over the concentration ranges of 0.025–15 and 0.014–1.67 *μ*g/mL for the* cis* and* trans *isomers, respectively.

The LLOQ was established using the signal-to-noise approach and defined as the analyte concentration giving a signal-to-noise ratio (S/N) of 5. Concentrations of analyte in plasma samples were determined by back calculation of the observed peak area ratios of analyte and IS from the best-fit calibration curve using a weighted (1/x^2^) linear regression.

Stability tests were conducted on the low and high QC samples for short-term (ambient temperature, 6 h), long-term (-80°C, 30 days), freeze/thaw (three freeze-thaw cycles), autosampler (ambient temperature, 12 h), and stock solution (4°C, 20 days) stability. If the bias of the tested QC samples was within ± 15% of their respective nominal concentrations, the samples were considered stable. Each analytical run consisted of a set of standard samples and a series of QC samples.

### 2.8. Application in a Pilot Pharmacokinetic Study

Four healthy male Chinese volunteers, aged 22.6 ± 2.2 years and weighing 62.87 ± 6.6 kg, were recruited for a single dose study. Blood samples were collected in heparin-treated tubes at 0, 0.125, 0.25, 0.5, 0.75, 1.0, 1.5, 2, 2.5, 3, 4, 6, 8, 10, 12, and 14 h after a dose of 500 mg cefprozil. Plasma samples were obtained by centrifugation of the blood samples at 3000 × *g* for 10 min and stored at -80°C. Plasma samples were thawed at ambient temperature before extraction as described above. Pharmacokinetic parameters for* cis-* and* trans-*cefprozil were estimated using the noncompartmental analysis function in Phoenix WinNonlin software, version 6.3 (Pharsight, Cary, NC, USA).

## 3. Results

### 3.1. Quantification and Separation

**Figure 3:**
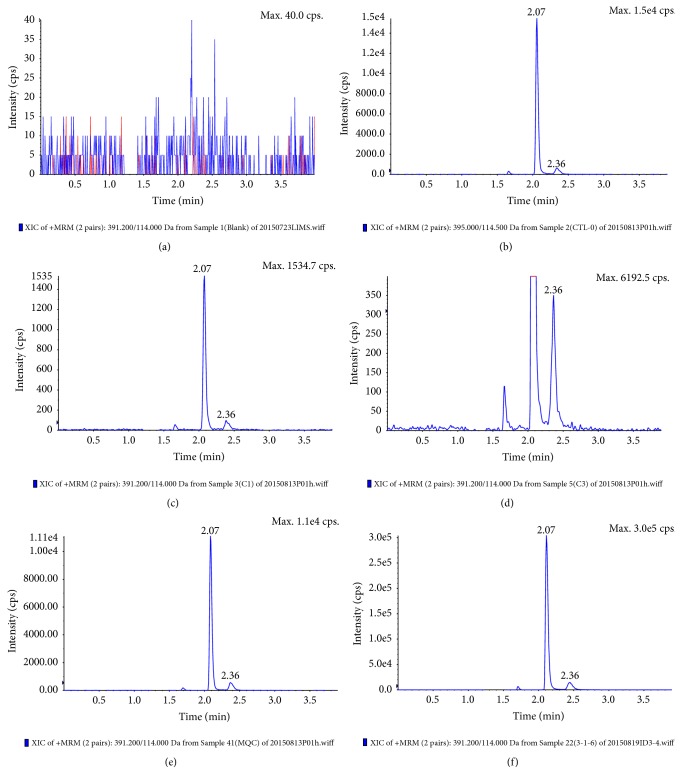
Chromatograms of (a) blank plasma, (b) plasma containing cefprozil-D4 (control-zero), (c)* cis*-cefprozil at the LLOQ (0.025 *μ*g/mL), (d)* trans*-cefprozil at the LLOQ (0.014 *µ*g/mL), (e) cefprozil at the moderate QC level (*trans*-cefprozil at 0.445 *μ*g/mL and* cis*-cefprozil at 4.1 *μ*g/mL), and (f) a plasma sample from a healthy Chinese volunteer (1.5 h after dosing).

Separation of the two cefprozil diastereomers was achieved using a C_18_ column with a mixture of acetonitrile and 0.5% formic acid as the mobile phase. The* cis* and* trans* isomers of cefprozil and IS were analyzed with a total run time of 4.0 min, giving retention times of 2.07 and 2.36 min for* cis*- and* trans*-cefprozil, respectively, as shown in Figure 3. Methanol was used for protein precipitation and cefprozil-D4 was used as the IS. Clean chromatograms were obtained and there was no significant matrix effect. The mass spectrometry conditions were optimized to obtain protonated molecules of cefprozil and IS. The collision energy was optimized to maximize the response of the fragment ion peak. The selected transitions were [M+H]^+^* m/z* 391.2→114.0 for cefprozil and 395.0→114.5 for IS.

### 3.2. Specificity

Results obtained from the analysis of six batches of blank plasma (Figure 3) were compared to those obtained from LLOQ plasma samples. The chromatograms indicated that there was no significant interference from endogenous plasma components.

### 3.3. Precision and Accuracy

Table 1 summarizes the accuracy and precision results. The accuracy is expressed as the relative error (RE) calculated for the QC sample analysis. The intra- and interassay precisions are expressed as coefficient of variance (CV). For each level (including QC and LLOQ samples), both intra*-* and interassay precisions for the* cis* and* trans* isomers of cefprozil were 2.0%–16.5%. The intra*-* and interassay accuracies for the isomers were between -7.1% and 6.0%. The results indicated that the developed method gave acceptable accuracy and precision.

### 3.4. Calibration Curves and LLOQ

The linearity of the calibration curves of the two isomers was calculated by plotting the peak area ratios (y) of* cis* isomers of analytes to* cis* isomers of IS and* trans* isomers of analytes to* trans* isomers of IS versus the nominal concentration (x) of cefprozil. The calibration curves were acquired by weighted (1/x^2^) linear regression analysis. Plasma calibration curves were determined in triplicate on three separate days to evaluate the linearity of the method. For each calibration curve, good linearity was observed over the concentration ranges of 0.025–15.0 *μ*g/mL and 0.014–1.67 *μ*g/mL for the* cis* and* trans* isomers, respectively. The correlation coefficient (r) values were 0.9995 (*cis* isomer) and 0.9973 (*trans* isomer). There was no significant change in the values of slope, intercept, or correlation coefficient in either inter- or intraday calibration curves. The RE values of the recalculated calibrators were found to be < 10.0%, which were sufficient for pharmacokinetic studies in human subjects. The LLOQ values were 0.025 *μ*g/mL (*cis *isomer) and 0.014 *μ*g/mL (*trans* isomer), as shown in Figure 3.

### 3.5. Recovery (Extraction Efficiency)

The extraction recovery of cefprozil was determined at low, medium, and high QC levels (0.05, 4.1, and 12.4 *μ*g/mL for the* cis* isomer; 0.028, 0.445, and 1.33 *μ*g/mL for the* trans* isomer) in sets of six replicates by comparing the responses from plasma samples spiked before extraction with those spiked after extraction. The extraction recoveries from human plasma were 96.2%–100.3% for the* cis* isomer and 96.9%–105.1% for the* trans* isomer (shown in Table 2). Recovery of the IS was 93% at a concentration of 1.15 *μ*g/mL.

### 3.6. Matrix Effect

The presence of endogenous plasma components in the samples could cause significant interference while utilizing an ESI source. The peak areas of the spiked samples after extraction were compared with the peak area of the spiked mobile phase to calculate matrix effects (ME). An ME value within 85%–115% indicates that there are no significant matrix effects, lower than 85% suggests ion suppression, and higher than 115% suggests ion enhancement. Two groups of samples were prepared at low and high QC concentrations of cefprozil. Group-1 was used to assess the MS/MS response of reference standard solutions. Reference standard solutions of cefprozil were diluted with mobile phase to the concentration expected in plasma spiked samples. Group-2 was composed of six randomly selected blank plasma samples spiked with reference standard solutions after extraction. The mean peak areas and RSD values were calculated for group-1 and group-2. ME was assessed by comparing the analytical results as follows: ME (%) = B/A × 100 (where A is the mean peak area of group-1 and B is the mean peak area of group-2). The results are shown in Table 2. The ME values for* cis*-cefprozil were 93.7% and 92.6% at two QC concentrations, while the ME values for* trans*-cefprozil were 92.4% and 91.6% at two QC concentrations.

### 3.7. Stability

Stability tests were conducted on the low and high QC samples as described in Section 2.7. Data from the stability experiments are presented in Table 3. The short-term stability was assessed at room temperature (controlled to within 20–25°C by air conditioning) for 6 h and the concentrations obtained were compared with those of the QC samples. The mean relative error was < 11.5% for both* cis* and* trans* isomers. Long-term stability was evaluated at -80°C for 30 days with a mean relative error < 10.0%. The deviation of freeze-thaw stability was < 6.7% of the nominal values for both* cis* and* trans* isomers. The mean relative error of autosampler stability for 12 h was < 8.9% of the nominal concentration. Finally, the stock solutions of cefprozil and IS were stable for 30 days at -20°C. The data showed that both the* cis* and* trans* isomers of cefprozil were stable under various storage/processing conditions and values were within ± 15% of the respective nominal concentrations.

### 3.8. Application in a Pilot Pharmacokinetic Study

**Figure 4:**
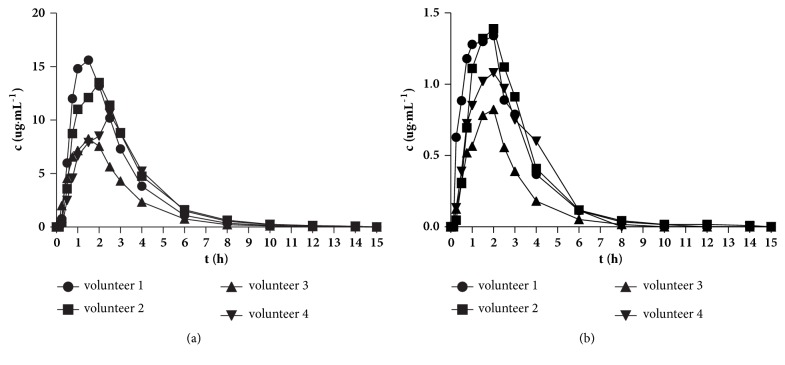
(a) The* cis*-cefprozil concentration-time curves and (b) the* trans*-cefprozil concentration-time curves from four healthy Chinese volunteers.

In a pilot pharmacokinetic study, the newly developed method was used successfully in healthy volunteers (Figure 4). The C_max_ values of* cis*- and* trans*-cefprozil were 12.9 ± 3.2 *μ*g/mL and 1.3 ± 0.2 *μ*g/mL, respectively. The T_max_ values of* cis*- and* trans*-cefprozil were 1.75 ± 0.5 h and 2.0 ± 0.35 h, respectively. Both diastereomers had similar t_1/2_ values of 1.87 and 1.66 h, respectively. The T_max_ and t_1/2_ values for cefprozil were similar to those previously reported in healthy Chinese volunteers [22], while the C_max_ values were a little higher than, but still similar to, those seen in Korean volunteers [12]. The AUC_0-t_ values were 42.4 ± 10.4 *μ*g·h/mL for* cis*-cefprozil and 3.85 ± 1.4 *μ*g·h/mL for* trans*-cefprozil in the current study, which were approximately 40% higher than those in a previous Chinese study, but similar to those in the Korean study [12].

## 4. Discussion

A number of methods to determine cefprozil using high-performance liquid chromatography have been reported, but there is still scope for improvement. Although some of the reported methods are able to detect cefprozil in a short time, these methods do not separate the* cis* and* trans* isomers of cefprozil [13–17]. Some methods are able to separate the* cis* and* trans* isomers of cefprozil but the analysis time for each sample is lengthy [12]. As the technology has developed, LC-MS/MS has been universally applied for analysis of multiple analytes and the method has improved sensitivity. Because cefprozil is composed of* cis* and* trans* isomers in an approximately 9:1 ratio, it is necessary to use a more sensitive method to detect the* trans* isomer. So far, the reported methods for detecting cefprozil isomers have used an electrospray ion source in negative ion mode [17, 22]. In the current study, we not only improved the HPLC method using an acetonitrile/water LC gradient, achieving baseline chromatographic separation of the isomers within 4 minutes, but also found greater sensitivity for determination of cefprozil in positive rather than negative ion mode. In this study, sample extraction required 100 *μ*L plasma, less than that required in the published methods, prepared by protein precipitation using methanol. Because less plasma is required in this method, smaller blood samples can be obtained from the patients.

## 5. Conclusion

A rapid, sensitive method for simultaneous determination of the* cis* and* trans* isomers of cefprozil was developed based on HPLC-MS/MS. Using an acetonitrile/water LC gradient, baseline chromatographic separation of the* cis* and* trans* isomers was achieved within 4 min. Compared to separate analysis of cefprozil diastereomers, simultaneous determination dramatically reduced the processing time for sample preparation and LC-MS/MS analysis. The method was successfully applied in a clinical study.

## 6. Compliance with Ethical Standards

The validated method was applied in a pilot pharmacokinetic study of cefprozil (dose of 0.5 g) in four healthy Chinese volunteers. The study was approved by the Research Ethics Committee of Guangdong General Hospital (Ethics Committee Document Number A201520) and informed consent was obtained from all subjects.

## Figures and Tables

**Table 1:** Intra- and interassay precision and accuracy of *cis*- and *trans*-cefprozil (*n *= 6).

Compound	Nominal concentration (*μ*g/mL)	Intra-assay Mean ± SD (*μ*g/mL)	Intra-assay Mean RE (%)	Intra-assay Precision (% CV)	Inter-assay Mean RE (%)	Inter-assay Precision (% CV)
*Cis-*cefprozil	0.025	0.026 ± 0.002	5.4	-0.1	3.0	9.2
	0.05	0.053 ± 0.002	4.4	3.0	6.0	4.4
	4.1	4.17 ± 0.09	2.0	0.3	1.7	3.3
	12.4	12.5 ± 0.56	3.1	1.3	0.8	9.9
*Trans*-cefprozil	0.014	0.013 ± 0.002	13.6	-6.9	-7.1	16.5
	0.028	0.028 ± 0.003	9.6	-0.5	0.1	5.1
	0.445	0.429 ± 0.036	6.9	-3.7	-3.8	14.3
	1.33	1.366 ± 0.098	7.0	2.7	3.0	8.5

SD: standard deviation; CV: coefficient of variance; RE: relative error.

**Table 2:** Assessment of recovery and matrix effect for *cis*- and *trans*-cefprozil (*n *= 6).

Compound	Concentration (*μ*g/mL)	Recovery (%) Mean ± SD	IS normalized matrix effect	
			Mean (%)	CV (%)
*Cis-*cefprozil	0.05	100.3±5.3	93.7	3.0
	4.1	96.2±3.0	NA	NA
	12.4	99.1±7.7	92.6	3.8
*Trans*-cefprozil	0.025	102.9±4.2	92.4	7.7
	0.445	96.9±4.3	NA	NA
	1.33	105.1±2.8	91.6	6.0

SD: standard deviation; CV: coefficient of variance; NA: not available.

**Table 3:** Stability of *cis*- and *trans*-cefprozil in human plasma under different storage conditions (*n *= 3).

Compound	Storage Conditions	Nominal Concentration (*μ*g/mL)	Mean RE (%)	CV (%)
*Cis-*cefprozil	Three freeze-thaw cycles	0.05	-0.7	2.4
		12.0	-4.7	0.6
	Stored at ambient temperature for 6 h	0.05	6.0	4.3
		12.0	2.1	2.4
	Stored in the auto-sampler at ambient temperature for 12h	0.05	-3.3	4.6
		12.0	0.9	6.5
	Plasma samples stored at -80°C for 30 days	0.05	-0.7	1.5
		12.0	3.3	1.1
*Trans*-cefprozil	Three freeze-thaw cycles	0.025	-6.7	3.2
		1.33	3.0	3.5
	Stored at ambient temperature for 6 h	0.025	5.7	5.4
		1.33	11.5	7.0
	Stored in the auto-sampler at ambient temperature for 12h	0.025	8.9	6.5
		1.33	2.1	7.0
	Plasma samples stored at -80°C for 30 days	0.025	-3.5	2.9
		1.33	10.0	6.3

CV: coefficient of variance; RE: relative error.

## Data Availability

The validation data used to support the findings of this study are available from the corresponding author upon request. The pilot pharmacokinetic study data used to support the findings of this study are currently under embargo while the research findings are commercialized. Requests for data, 6 months after publication of this article, will be considered by the corresponding author.
